# On the Most Frequented Watering Places on the Continent

**Published:** 1837-01

**Authors:** 


Art. V.—*An Account of the most-frequented Watering Places on the Continent, and of the Medicinal Application of their Mineral Springs; with Tables of Analysis, and an Appendix on English Mineral Waters.* By Edwin Lee, Esq. m.r.c.s.—*London,* 1836. Small 8vo. pp. 232.

This little volume is by the author of the work on the Medical Institutions of the Continent, of which we gave an account in our second Number; but, although it bears the same impress of industry and good sense, it is not equal to its predecessor. In the present want, however, of any other book in our language which gives a general account of the continental mineral waters, this little work cannot fail to be serviceable to invalids who travel abroad; and it will likewise be useful as a convenient remembrancer to physicians in this country who may be consulted by their patients on the eve of their departure. It gives a concise, and we doubt not accurate, analysis of all the principal waters, and a brief description of most of the places where they are situated. These analyses and descriptions seem for the most part taken from the local guide-books, and from the pamphlets by the resident physicians on the virtues of their respective springs, which abound so remarkably on the continent. We do not quarrel with the author for availing himself of these sources of information, no doubt the best open to him as a traveller; but we must guard our readers against expecting in the present work any thing like original views, or instructions sufficiently ample and discriminating to enable the patient to direct his own proceedings on arriving at the source to which he may be directed. Even from the guide-books the author might have selected many useful things which he has omitted. He ought certainly to have given more frequently than he has done the usual doses and most ordinary mode of using the different waters.

An English treatise on the general subject of mineral waters, their true medicinal value, their mode of acting on the system, and the proper method of applying them in the proper cases, is yet a desideratum in our literature; and we think that it reflects little credit on the physicians of our various watering places—both in past and present times—that such a work has yet to be written. It is true our literature is not destitute of works of this kind; but we hold it to be altogether destitute of works worthy of the subject, or fit to come in competition with the many learned and profound treatises on this branch of medicine which exist in other languages, more particularly the German. Indeed, we do not hesitate to say that, taking the profession generally in this country, there is not one branch of therapeutics in which so little is really known as in that which relates to the use of mineral waters. The dominion of the *medicina perturbatrix* formerly almost universally prevalent in our medical schools, taught us to disregard all means which do not manifest their agency by some powerful effect on the system ; and the gross views thence derived have led too many of our physicians to look upon the most valuable mineral waters as mere forms of cathartics, and, hence, in almost all cases in which they are employed, to interpose the use of mercurials and other purgatives. Nothing can be more inaccurate than such views, and nothing worse than such practice ; and we are disposed to derive from this source the explanation of the fact, that the value of mineral waters, in many chronic diseases, is extremely underrated in this country. We observe, by the date of Mr. Lee's preface, that he has taken up his abode at Cheltenham. May we express a hope that he carries to that fashionable place of resort for patients, a determination to study the medicinal action of its waters on the principles of a sounder philosophy than seems to have guided most of his predecessors who have published books concerning them? If he does not, we shall say that he has derived less advantage from his visits to the mineral springs of the continent than he might have done.

